# A Rare Case of Idiopathic Gonadal Vein Thrombosis

**DOI:** 10.7759/cureus.21323

**Published:** 2022-01-17

**Authors:** Hany A Zaki, Haris Iftikhar, Ahmed E Shaban, Omar Khyatt, Eman E Shaban

**Affiliations:** 1 Emergency Medicine, Hamad Medical Corporation, Doha, QAT; 2 Internal Medicine, Mansoura General Hospital, Mansoura, EGY; 3 Radiology, Hamad Medical Corporation, Doha, QAT; 4 Cardiology, Al Jufairi Diagnosis and Treatment, Doha, QAT

**Keywords:** case report, idiopathic, imaging, ovarian vein thrombosis, postpartum, pregnancy, gonadal vein thrombosis, deep vein thrombosis (dvt)

## Abstract

Gonadal vein thrombosis, also known as ovarian vein thrombosis, is a rare medical condition presenting mostly in the postpartum period. Gonadal vein thrombosis is associated with conditions such as inferior vena cava thrombosis, sepsis, and pulmonary emboli which can lead to high morbidity and mortality. This report illustrates the case of a 25-year-old female patient who presented to the emergency department with a history of abdominal pain for over three days. The pain initially started centrally for a day, gradually, without radiation, and then became more localized in the right lower area and radiated to the genital area. The patient had a history of a cesarean section two years ago. Based on the presentation, an abdominal computed tomography scan demonstrated thickened tortuous right gonadal vein with the possibility of right gonadal vein thrombophlebitis and thrombosis. Having been examined by the general surgery and gynecology teams, a treatment plan was drawn involving thrombophilia workup and therapeutic anticoagulation. Although a rare entity, idiopathic gonadal vein thrombosis can present in the emergency department with vague abdominal pain and unclear diagnosis with a lack of risk factors. A high index of suspicion and imaging might be helpful to make this unique diagnosis.

## Introduction

The ovaries are paired structures weighing 4-8 g (0.14-0.3 ounces) and are linked with the uterus and the lateral pelvic wall by ligaments. Gonadal vein thrombosis is mainly a puerperal complication. It is a rare form of deep vein thrombosis that can be fatal. The vein of the right ovary is associated with the site of thrombosis because the right ovarian veins are larger than the left, as well as due to retrograde flow in right veins caused by a lack of competent valves. The right ovarian vein connects to the inferior vena cava (IVC) just below the left renal vein, while the vein of the left ovary empties into the left renal vein. It is worth mentioning that the right ovarian vein connects to the IVC at an acute angle, thus increasing its susceptibility to compression. A primary risk factor for ovarian vein thrombosis (OVT) is pregnancy. This is attributed to the stagnation of blood within the dilated vein of the ovary combined with contractions occurring during cesarean section (CS) or natural delivery [1]. CS is a major contributor to OVT [2]. OVT is prevalent in 0.05-0.18% of pregnancies globally, with its incidence increasing to 1-2% following CS [3]. The results from a prospective study showed that CS contributed to a 0.1% incidence of OVT [4].

Gonadal vein thrombosis may cause the thrombosis to propagate into the IVC. IVC thrombosis is a manifestation of deep venous thrombosis and is a primary risk factor for pulmonary embolism [5]. As such, thrombosis of the IVC and ovarian vein is the major cause of maternal mortality globally [6]. Computed tomography (CT) can be used to diagnose thrombosis of the IVC and ovarian vein. Additionally, fibrinogen, D-dimer, and blood fibrin degradation product levels can be used as markers of the rapid progression of fibrinolysis and blood coagulation. D-dimer plays a crucial role as a first-line screening test for venous thromboembolism diagnosis [7]. Early diagnosis and appropriate treatment can help prevent these potentially fatal complications [8]. In this report, we present a rare case of gonadal vein thrombosis diagnosed incidentally in a patient with abdominal pain by radiological imaging.

## Case presentation

A 25-year-old female patient presented to the emergency department with a history of abdominal pain for over three days. The pain initially began approximately one day ago, without radiation, and then became more localized in the right lower quadrant while radiating to the genital area. In addition, the patient reported a burning sensation while urinating, as well as nausea without vomiting. She denied having any fever, vomiting, anorexia, or diarrhea. There was no chest pain, dizziness, or shortness of breath. There was no previous episode of a similar complaint.

Before arriving at the emergency department, the patient had consulted a private clinic where an ultrasound (USG) was performed. USG showed a blind-ending, non-compressible bowel loop in the right inferior fossa with a diameter of 3.5 × 1.4 mm. Moreover, mild hyperemia was noted on color Doppler along with adjacent mild inflammatory changes in the form of omental hyperechogenicity. The patient was informed that she had urinary tract infections (UTIs).

The patient had previously undergone a CS two years ago. Her last menstrual period was one week ago with regular periods. There were no known comorbidities. Her family and social history were unremarkable.

On physical examination, the patient appeared well. Her initial vitals include a heart rate of 78 beats per minute, a temperature of 36.8°C, oxygen saturation of 99% on room air, and blood pressure of 110/67 mmHg. Abdominal examination revealed right lower quadrant tenderness but no rebound tenderness. There was no guarding or rigidity. She had normal bowel sounds, and the rest of her examination was unremarkable.

**Figure 1:**
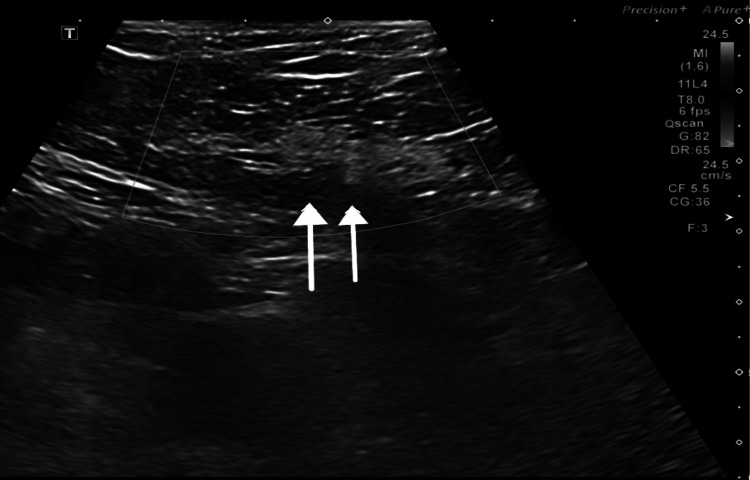
Ultrasound of the abdomen showing (white arrows) oval-shaped hyperechogenic area within the abdominal wall, non-vascular on color Doppler, suggesting fat necrosis or lipoma.

Her laboratory blood investigations were unremarkable. Her USG was repeated in our emergency department. It showed a small non-specific hyperechoic area within the abdominal wall (Figure 1).

**Figure 2:**
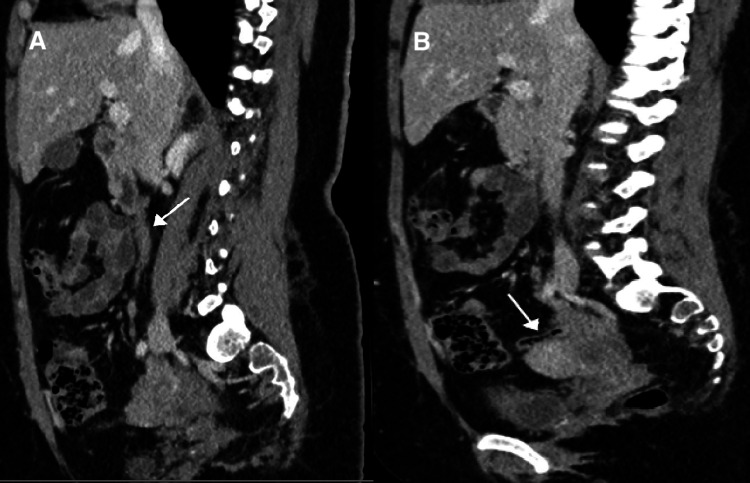
(A) Sagittal CT of the abdomen (venous phase) showing thickened tortuous right ovarian vein (white arrow). (B) Sagittal CT of the abdomen (venous phase) showing normal-looking appendix (white arrow). CT: computed tomography

Thereafter, a CT of the abdomen was performed which showed a normal-looking appendix (Figure 2).

**Figure 3:**
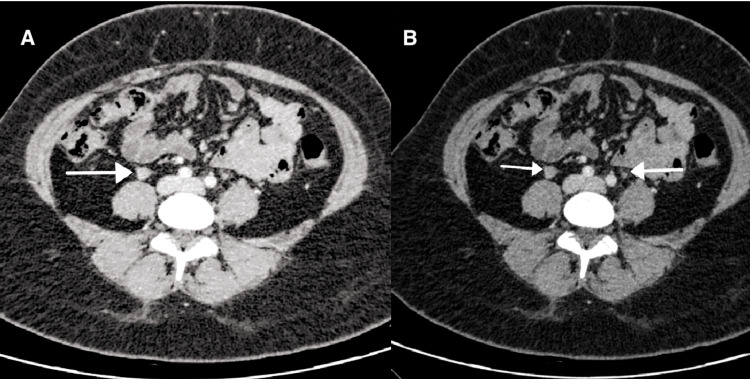
(A) CT scan (axial section, venous phase) showing dilated right ovarian vein anterior to the psoas muscle with subtle stranding (arrow). (B) CT scan (axial section) showing thickened right gonadal vein compared to the left (arrows). CT: computed tomography

**Figure 4:**
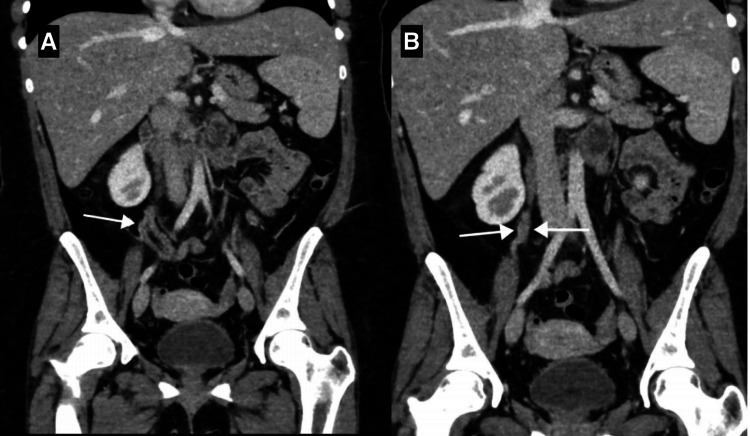
(A) CT scan (coronal view, porto-venous phase) showing dilated tortuous right ovarian vein with subtle stranding, suggesting thrombosis (white arrow). (B) CT scan (coronal view, venous phase) showing dilated right ovarian vein (white arrows). CT: computed tomography

There was no evidence of colitis. There was a thickened tortuous right gonadal vein draining into the IVC. It appeared thickened compared to the left and tortuous with subtle fat stranding. Although there was no obvious major filling defect, these findings suggested OVT (Figures 3, 4).

The patient was seen and cleared by the general surgery and gynecology teams. She was admitted by the medical team for thrombophilia and malignancy workup and therapeutic anticoagulation. She was started on apixaban. Her thrombophilia workup and tumor markers were negative. She was discharged on anticoagulation for three months with further follow-up with internal medicine and gynecology teams.

## Discussion

The first case of postpartum OVT was reported in 1956 [9]. Several studies have reported on this rare medical condition. However, idiopathic OVT has been less frequently reported in the literature in a few case reports.

Virchow’s triad is utilized for a pathophysiological explanation of OVT. It is used because of the association between pregnancy and a hypercoagulable state, venous stasis attributed to uterine compression of the IVC, and endothelial trauma during child delivery. Local inflammation may also be a cause in some cases. OVT has an estimated incidence of 0.05-0.18% of pregnancies, with the most affected patients aged between 30 and 40 years. The right ovarian vein is affected in 80-90% of cases, usually within 2-15 days after delivery. The risk of thrombosis increases by 1-2%, with multiparity identified as a major general risk factor for thrombosis [10,11]. Rare causes of OVT include malignancies, pelvic inflammatory disease, pelvic surgical procedures, and Crohn’s disease [12]. In the case of malignant tumors, especially those subjected to chemotherapy, there may be a risk of developing OVT; however, it is usually asymptomatic and the thrombus does not need any treatment to resolve [12].

Reported risk factors for OVT include hypercoagulation conditions such as antiphospholipid syndrome, systemic lupus erythematosus, presence of factor V Leiden, hyperhomocysteinemia, hemoglobinuria, deficiency of protein C and S, and heparin-induced thrombocytopenia [13,14]. Our case is unique as the patient did not have these risk factors and rare causes, as described above. The incidence of idiopathic OVT has not been reported in the literature. Basit et al. reported a case of idiopathic bilateral OVT. Their literature review identified 12 cases of idiopathic unilateral OVT, most of which had risk factors for OVT [15].

The signs and symptoms associated with OVT include lower flank or abdominal pain, leukocytosis, and fever, usually within the first week after delivery. An uncommon characteristic co-existence is OVT along with obstruction of the right ureter and hydronephrosis. Regarding the anatomy, the right ovarian vein crosses before the right ureter at the L4 vertebral level as it approaches the IVC [16].

Magnetic resonance imaging, CT scans, and USG can be used for diagnostic imaging. Magnetic resonance angiography (MRA) is the most specific and most sensitive diagnostic modality. However, MRA is reserved for critical situations while other techniques are commonly considered due to speed and cost [17].

The diagnostic dilemma is attributed to the rarity of this medical entity. Acute appendicitis where lower abdominal pain is the primary symptom cannot be excluded, usually resulting in an acute appendectomy.

Antibiotics and anticoagulation are the primary treatment for gonadal vein thrombosis. Morbidity is due to complications including sepsis, thrombus extension to the renal veins and IVC, and pulmonary embolism. Gonadal vein thrombosis has a mortality as high as 5% mostly caused by pulmonary embolism, which has been reported to have a 13.2% incidence [18]. If the patient is unresponsive to standard medical treatment or there are severe complications, treatment options include hysterectomy, placement of IVC Greenfield filter, thrombectomy, or ligation of the significantly injured infrarenal IVC [19]. Prophylaxis is not recommended during subsequent pregnancies unless the clinician can prove the existence of a hypercoagulable state.

## Conclusions

Although a rare entity, patients with idiopathic gonadal vein thrombosis can present in the emergency department with vague abdominal pain and unclear diagnosis with a lack of risk factors. A high index of suspicion and a low threshold for imaging might be helpful to make this unique diagnosis. OVT should be a part of the differential diagnosis for postoperative women presenting with abdominal symptoms considering the high fatality associated with OVT. Multiple complications such as sepsis, thrombus extension, and pulmonary embolism can manifest with OVT in the puerperal setting, thus supporting anticoagulation therapy.
